# Complete Revascularization in NSTE-ACS and Multivessel Disease: Clinical Outcomes and Prognostic Implications

**DOI:** 10.3390/life15081299

**Published:** 2025-08-15

**Authors:** Silviu Raul Muste, Cristiana Bustea, Elena Emilia Babes, Francesca Andreea Muste, Gabriela S. Bungau, Delia Mirela Tit, Alexandra Georgiana Tarce, Andrei-Flavius Radu

**Affiliations:** 1 Doctoral School of Biomedical Sciences, Faculty of Medicine and Pharmacy, University of Oradea, 410087 Oradea, Romania; muste.silviuraul@student.uoradea.ro (S.R.M.); furko.francescaandreea@student.uoradea.ro (F.A.M.); gbungau@uoradea.ro (G.S.B.); dtit@uoradea.ro (D.M.T.); andreiflavius.radu@uoradea.ro (A.-F.R.); 2 Department of Preclinical Disciplines, Faculty of Medicine and Pharmacy, University of Oradea, 410073 Oradea, Romania; 3 Department of Medical Disciplines, Faculty of Medicine and Pharmacy, University of Oradea, 410073 Oradea, Romania; 4 Department of Pharmacy, Faculty of Medicine and Pharmacy, University of Oradea, 410028 Oradea, Romania; 5 Faculty of Medicine and Pharmacy, University of Oradea, 410073 Oradea, Romania; tarce.alexandrageorgiana@student.uoradea.ro; 6 Department of Psycho-Neurosciences and Recovery, Faculty of Medicine and Pharmacy, University of Oradea, 410073 Oradea, Romania

**Keywords:** acute coronary syndrome, NSTE-ACS, multivessel coronary disease, complete revascularization, STEMI, left ventricular ejection fraction

## Abstract

Non-ST-segment-elevation acute coronary syndrome (NSTE-ACS) often coexists with multivessel coronary artery disease (MVD), complicating treatment decisions. Current guidelines suggest complete revascularization (CR), yet robust evidence in hemodynamically stable patients remains insufficient. However, the comparative benefit of CR over incomplete revascularization (IR) in reducing ischemic events and improving cardiac function in this population is not well established. The aim of this study was to evaluate the impact of CR on all-cause mortality, cardiac death, and ischemic readmissions at 6 and 12 months, as the composite primary outcome, and to assess left ventricular ejection fraction (LVEF) improvement at discharge and hospital length of stay, as secondary outcomes. A total of 282 hemodynamically stable NSTE-ACS patients with MVD were included, of whom 218 (77.3%) underwent CR and 64 (22.7%) IR. The primary composite outcome occurred in 40.6% of IR patients versus 11.0% in the CR group at 6 months (*p* < 0.001), and 68.8% vs. 22.0% at 12 months (*p* < 0.001). CR was associated with significantly lower rates of all-cause and cardiac death, myocardial infarction, and unstable angina. Stroke incidence was similar. Event-free survival favored CR. Multivariable analysis identified CR and baseline LVEF as independent predictors of 12-month outcomes (HR for CR: 7.797; 95% CI: 3.961–15.348; *p* < 0.001; HR for LVEF: 0.959; CI: 0.926–0.994; *p* = 0.021). These findings strongly support CR as the preferred therapeutic strategy. Future prospective randomized studies are warranted to confirm the results.

## 1. Introduction

Non-ST-segment-elevation acute coronary syndromes (NSTE-ACS) encompass a spectrum of clinical conditions resulting from the rupture of an atherosclerotic plaque, leading to intermittent or incomplete thrombotic occlusion of the artery responsible for the infarction [1]. Atherosclerosis, the underlying cause, is a chronic inflammatory disease, and inflammation plays a central role in plaque progression and destabilization [2]. Current clinical guidelines emphasize the importance of risk stratification when deciding on revascularization strategies for NSTE-ACS patients. An early invasive approach is recommended for very high-risk cases, defined by factors such as hemodynamic instability, ongoing myocardial ischemia, cardiac arrest, or life-threatening arrhythmia. For high-risk patients, revascularization is advised within 24 h, based on confirmed diagnoses, high GRACE risk scores, or dynamic ECG changes. In contrast, a less urgent inpatient strategy is suitable for non-high-risk patients [3].

Patients with NSTE-ACS are often older and present with multiple comorbidities, such as diabetes mellitus, chronic kidney disease (CKD), or previous cerebrovascular events. Additionally, coronary angiography frequently reveals multivessel disease (MVD) in this population, making revascularization decisions particularly challenging. Identifying the infarct-related artery can be difficult based on angiographic findings alone, and prolonged dual antithrombotic therapy carries potential risks [4].

The presence of MVD has been linked to less favorable clinical outcomes when contrasted with isolated single-vessel involvement [5,6,7]. The European Society of Cardiology (ESC) guidelines [3] recommend complete revascularization (CR) for NSTE-ACS patients with MVD (Class IIa, Level C), a decision supported by observational studies and meta-analyses [8,9]. Although multiple randomized clinical trials (RCTs) have investigated the outcomes of addressing non-culprit lesions in individuals diagnosed with ST-segment-elevation myocardial infarction (STEMI) [10,11], there is a notable lack of RCTs specifically addressing this strategy in non-ST-elevation myocardial infarction (NSTEMI) patients. To date, no randomized clinical trials have definitively assessed the benefits of treating significant non-culprit lesions alongside the culprit artery identified by coronary angiography in NSTEMI setting.

Previous trials, such as the BIOVASC and SMILE studies, have focused on the timing of revascularization in patients with acute coronary syndromes, specifically those with NSTE-ACS and MVD. The BIOVASC randomized trial [12], which included patients with acute coronary syndromes (both STEMI and NSTEMI), demonstrated in its sub-study focused on non-ST-segment-elevation patients that immediate CR of the culprit artery was associated with a lower risk of the primary composite outcome, myocardial infarction, and unplanned ischemia-driven revascularization, compared to staged CR. Similarly, the SMILE trial [13], which enrolled patients with NSTEMI and multivessel coronary disease, showed that single-stage percutaneous coronary intervention (PCI) during the index procedure was superior to multistage PCI during the index hospitalization in reducing the incidence of major adverse cardiovascular and cerebrovascular events. These findings suggest potential benefits of single-stage revascularization strategies in improving outcomes. However, the question of whether revascularization of non-culprit lesions in patients with NSTE-ACS confers a definitive clinical benefit remains unresolved [11,12].

Moreover, practical considerations frequently lead to incomplete revascularization (IR) in clinical settings. These include lesion complexity, diffuse disease, patient frailty, risk of contrast nephropathy, and operator judgment [14,15]. Despite its frequency, the consequences of IR on both short- and long-term clinical outcomes in NSTE-ACS remain poorly characterized. Beyond ischemic outcomes, other clinically meaningful endpoints deserve attention, particularly in high-risk NSTE-ACS patients. These include improvement in left ventricle ejection fraction (LVEF), and duration of hospital stay, all of which impact recovery, resource utilization, and quality of life [15,16]. These outcomes may not solely depend on epicardial vessel patency, as inadequate microvascular reperfusion, known as the no-reflow phenomenon, can still occur despite successful revascularization [17].

This investigation addresses this gap by evaluating, firstly, the impact of CR on all-cause death, cardiac death and readmissions due to ischemic events (i.e., myocardial infarction, unstable angina, or stroke) at six months and one year and, secondly, to assess the improvement of ejection fraction at discharge and the hospitalization lengths among hemodynamically stable NSTE-ACS patients with MVD.

## 2. Materials and Methods

### 2.1. Study Design

This study is a single-center retrospective observational cohort study conducted at Bihor County Emergency Clinical Hospital, a tertiary-care cardiac center. Medical records of patients admitted in the Cardiology Department with a diagnosis of NSTE-ACS between January 2021 and December 2023 were reviewed. The study was approved by the Institutional Review Board (Hospitals’ Ethics Commission number 31129/11.10.2024), with a waiver of informed consent due to the retrospective nature of the research.

### 2.2. Population Under Study

Participants were deemed eligible for enrollment based on the inclusion criteria outlined in Table 1, which also details the exclusion criteria.

Patients were divided into two groups based on revascularization strategy: CR group: patients who underwent PCI to all angiographically significant lesions in ≥2 vessels during index hospitalization, and IR group: patients who received PCI to the culprit vessel only, without intervention on other significant lesions.

Data were extracted from the institutional electronic medical record system and catheterization laboratory database. The following variables were collected: baseline characteristics (i.e., age, sex, presence of diabetes mellitus, essential hypertension, hypercholesterolemia, CKD, atrial fibrillation, smoking status, blood pressure and heart rate at admission, troponin level at admission, baseline serum creatinine, and baseline LVEF, GRACE risk score was also calculated), procedural details (i.e., the culprit lesion location, type of approach, and antiplatelet regimen after PCI), follow-up data (i.e., all-cause death, cardiac death, ischemic events (i.e., MI, unstable angina or stroke) at 6 months and 1 year, LVEF at discharge and hospital length of stay). LVEF was assessed using two-dimensional echocardiography (Phillips CX50 ultrasound system, manufactured by Phillips Ultrasound, Bothell, WA, USA, with partial assembly in China, 2019) by the Simpson method, and it was reported as a percentage. The improvement in LVEF was calculated by comparing baseline and discharge values.

Coronary angiography was performed with an Azurion 3 System (Philips Medical systems Nederland B.V, Best, The Netherlands, 2020). Regarding the angiographic analysis, coronary lesions were visually evaluated by two experienced interventional cardiologists. To facilitate identification of the culprit lesion, the angiographic data were analyzed in correlation with ECG (Nihon Kohden ECG-3350 machine manufactured by Nihon Kohden, Tokyo, Japan, 2009) changes and ventricular wall motion abnormalities observed on echocardiography. Identification of the culprit artery was based on several angiographic features, including impaired blood flow, intraluminal filling defects indicative of thrombus, and acute occlusions characterized by abrupt, squared, or convex proximal endings. Additionally, the presence of intraluminal filling defects within patent vessels adjacent to stenotic regions with uniform contrast opacification was considered. Plaque ulceration was defined by contrast extravasation and hazy vessel contours extending beyond the lumen, while plaque irregularity involved uneven margins or overhanging edges. Arterial dissection was also included among the diagnostic criteria [18]. The revascularization procedure was considered successful when the final residual stenosis was below 50% accompanied by TIMI grade 3 flow [19].

The primary outcome was a composite of all-cause death, cardiac death [18] (as adjudicated via clinical notes, discharge diagnoses, telephone contact, and death certificates where available), and ischemic readmissions (defined as hospitalizations due to myocardial infarction, unstable angina, or stroke). Time points for analysis included cumulative incidence at 6 months and 1-year post-PCI. The secondary outcomes were as follows: the incidence of each event from the composite primary outcome at 6 months and 1-year post-PCI, change in LVEF from baseline to discharge, and hospital length of stay (in days).

### 2.3. Statistical Evaluations

The statistical methods applied for data analysis are detailed in Table 2.

## 3. Results

A total of 282 hemodynamically stable NSTE-ACS patients with multivessel disease were included in the analysis. Of these, 218 patients (77.3%) underwent CR, while 64 patients (22.7%) received IR. Baseline demographic and clinical characteristics are summarized in Table 3.

Baseline characteristics (Table 3) were generally balanced, with no significant differences in parameters like age, presence of hypercholesterolemia, atrial fibrillation at presentation, and GRACE risk score. However, the IR group had a significantly higher prevalence of male patients (66.05% vs. 53.12%, *p* = 0.01), hypertension, diabetes mellitus, and CKD. In this study, data on CKD were collected irrespective of its specific stages. Patients were classified based on the presence or absence of CKD.

Regarding the location of the culprit lesion, only the right coronary artery (RCA) was involved significantly more frequently in IR patients (Table 4). There was no difference between the two groups concerning the arterial access. Clopidogrel was used mostly in the IR group, while ticagrelor was mainly used in CR patients.

At 6 and 12 months, the IR group demonstrated significantly higher rates of adverse clinical events (Table 5). The primary composite outcome occurred in 40.6% of IR patients vs. 11.0% of CR patients at 6 months (*p* < 0.001), and 68.8% vs. 22.0% at 12 months (*p* < 0.001). IR was also associated with increased all-cause mortality at both 6 months and 12 months. Similarly, cardiac death was significantly higher in IR patients at both time points. Rates of non-fatal events, comprising unstable angina and myocardial infarction, were markedly increased in the IR group. Stroke incidence did not differ significantly between groups.

LVEF at discharge was lower in the IR group, and improvement in LVEF from admission to discharge was significantly lower in the IR group. There was no significant difference in hospital length of stay between groups (Table 6).

**Figure 1:**
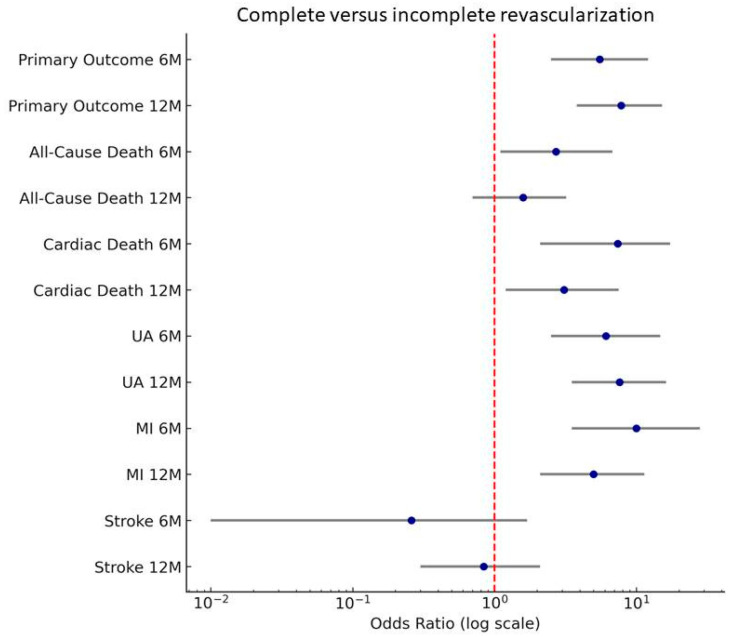
Forest plot for the composite primary outcome and for each event from the composite primary outcome. Primary outcome, composite of all-cause death, cardiac death, and ischemic readmissions due to unstable angina, myocardial infarction, stroke; UA, unstable angina; MI, myocardial infarction.

Forest plot analysis illustrates the significantly increased odds of adverse outcomes in the IR group (Figure 1). Primary outcome at 12 months, myocardial infarction at 6 months, and cardiac death at 6 months showed the highest risk of occurrence. Stroke rates did not differ significantly.

Regarding the factors associated with primary outcome at 12 months, worse outcomes were significantly associated with lack of CR, female sex, hypertension, diabetes mellitus, lower LVEF at admission and discharge, and use of clopidogrel (Table 7).

**Figure 2:**
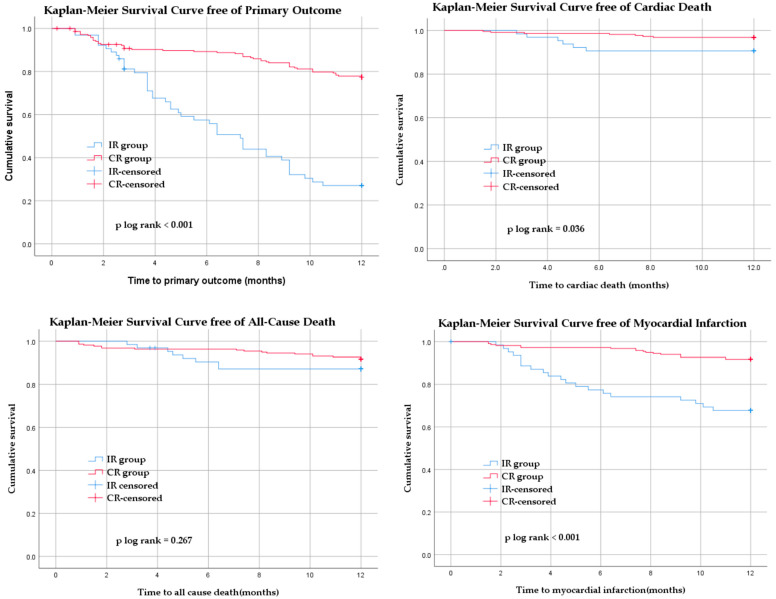
Kaplan–Meier event-free survival curves at 12 months for primary outcome, cardiac death, all-cause death, myocardial infarction. CR, complete revascularization; IR, incomplete revascularization; primary outcome, composite of all-cause death, cardiac death, and ischemic readmissions due to unstable angina, myocardial infarction, or stroke.

To evaluate the direct, unadjusted relationships between CR and clinical outcomes, bivariate correlation analysis was performed. The presence of CR was significantly and inversely correlated with adverse cardiovascular events at both 6 and 12 months. Specifically, CR was negatively correlated with the primary outcome at 6 months (r = −0.325, *p* < 0.001) and 12 months (r = −0.418, *p* < 0.001). Similarly, negative correlations were found between CR and unstable angina at 6 months (r = −0.245, *p* < 0.001) and 12 months (r = −0.351, *p* < 0.001), as well as myocardial infarction at 6 months (r = −0.312, *p* < 0.001) and 12 months (r = −0.282, *p* < 0.001). No significant correlation was observed between CR and stroke at either time point (6 months r = 0.08, *p* = 0.18; 12 months r = 0.01, *p* = 0.82) (Figure 2).

These findings suggest that patients who underwent CR experienced significantly fewer ischemic events, even before adjusting for potential confounding factors.

Event-free survival was higher in the CR group, as shown in the Kaplan–Meier plot (Figure 2). Multivariable logistic regression identified IR and reduced LVEF at admission as independent predictors of the 12-month primary outcome. After adjusting for covariates, CR was associated with a significantly lower hazard of experiencing the composite event (HR = 7.797, 95% CI: 3.961–15.348, *p* < 0.001). A reduced LVEF at admission was also independently associated with a higher incidence of the primary outcome (HR = 0.959, 95% CI: 0.926–0.994, *p* = 0.021). Other covariates (i.e., sex, hypertension, diabetes, troponin level, LM involvement in culprit lesion location, and clopidogrel use) were not statistically significant. The model demonstrated acceptable fit (Hosmer–Lemeshow *p* = 0.136) and classified outcomes with 74.8% overall accuracy. Kaplan–Meier survival analysis confirmed significantly poorer survival free from cardiac death in the IR group (Log-rank *p* = 0.001 at 6 months, *p* = 0.036 at 12 months). No statistically significant differences were observed for all-cause mortality (although there was a trend toward significance at 6 months, Log-rank *p* = 0.001).

## 4. Discussion

The present retrospective study examined the impact of CR versus IR in hemodynamically stable patients presenting with NSTE-ACS and multivessel coronary artery disease. Our findings suggest that CR was associated with significantly better clinical outcomes at both 6 and 12 months. Patients in the CR group experienced fewer major adverse cardiovascular events (MACE), including MI, UA, and cardiac death, and demonstrated significantly greater improvement in LVEF. The bivariate correlations further support the benefit of CR, demonstrating consistent negative associations between CR and MI, UA, and composite outcomes. Patients in the IR group had a higher prevalence of traditional risk factors such as diabetes, hypertension, and CKD, which may have influenced both the feasibility of CR and the worse outcomes observed. Despite these baseline imbalances, multivariate analysis confirmed CR as an independent predictor of reduced adverse events. Hospital length of stay was not significantly different between groups. These findings underline the potential benefits of addressing all significant lesions, thereby reducing ischemic burden, and improving long-term outcomes.

Our results align with prior observational studies suggesting a potential benefit of CR in NSTE-ACS patients. For example, improved outcomes in terms of MACE have been reported with multivessel PCI [21]. A systematic review by Faro et al. [22], analyzing randomized controlled trials and high-quality observational studies, demonstrated that CR in ACS patients with MVD was associated with a significant reduction in MACE, particularly when the intervention occurred during the index hospitalization. While this review predominantly focused on broader ACS populations, our findings narrow the scope to hemodynamically stable NSTE-ACS patients and suggest a similar benefit, especially regarding ischemic readmission and early improvement in ejection fraction.

Expanding on this, Bianchini et al. [23] performed a meta-analysis of over 180,000 patients and found that CR in NSTE-ACS patients significantly reduced all-cause mortality and recurrent MI. These results reinforce the validity of our composite outcome (i.e., cardiac death and ischemic readmissions), providing strong external support for the clinical benefit of full revascularization in our study population.

In the e-ULTIMASTER registry, a global observational study that enrolled 37,198 patients, an analysis by Jiménez Díaz et al. [24], showed that CR in NSTE-MI patients was independently associated with a lower risk of mortality and MACE compared to incomplete revascularization (IR) at both 3-month and 1-year follow-up.

Manuca et al. [25] provided a contemporary review on the management of non-culprit lesions in multivessel coronary disease. They emphasized the importance of individualized strategies based on plaque vulnerability, physiological assessment, and timing. While our study does not include FFR or imaging data, our results are aligned with the principle that addressing non-culprit stenoses may translate into clinical benefits in stable patients.

In a cohort analysis, Baumann et al. [26] reported that a significant proportion of NSTEMI patients with MVD are still managed with culprit-only PCI, often due to perceived procedural complexity or risk. Our study highlights that, when feasible, CR can be accomplished without extending hospital stay.

Pandit et al. [5] also compared target-vessel and CR strategies in NSTE-MI without shock. They reported improved outcomes in the CR arm, particularly with respect to recurrent ischemia. These findings are comparable to our data, which showed a lower rate of ischemic readmissions in patients who underwent more comprehensive intervention.

Further supporting the mortality benefit of complete PCI, Ahmad et al. [27] conducted an updated meta-analysis of randomized trials involving STEMI patients. Although our study focused on NSTE-ACS, the observed consistency in benefit across ACS subtypes suggests a class effect favoring CR, irrespective of initial ST-segment presentation.

The greater improvement in LVEF observed in the CR group may reflect the recovery of hibernating myocardium following revascularization of non-culprit vessels. This is consistent with prior imaging studies showing improved myocardial perfusion and function post-complete PCI [28]. Dahroug et al. [29] used speckle-tracking echocardiography to demonstrate improved left ventricular recovery post-total revascularization in NSTEMI patients. Our results similarly revealed a more favorable improvement in ejection fraction at discharge in the CR group, suggesting an early positive impact on myocardial function.

Moreover, insights from the OPTIMA-2 randomized trial, as reported by Fagel et al. [30], showed an improvement of LVEF at 30-day follow-up, among NSTEMI patients with CR, regardless of the timing of revascularization strategy (direct strategy <3 h or an early strategy 12–24 h) Although our study was retrospective, the trend toward better left ventricular function in the complete group may be partially explained by early comprehensive ischemic relief.

The CORALYS registry subanalysis by Bruno et al. [31] showed that CR in ACS patients was independently associated with a reduced incidence of heart failure. Interestingly, the results were consistent in patients with LVEF > 40%, while no benefit was observed in patients with LVEF < 40%. These findings are particularly compelling given that improvement in ejection fraction is a key predictor of long-term prognosis.

In contrast, other studies, have shown conflicting results, particularly when applied to the higher-risk NSTE-ACS subgroup with comorbidities such as renal impairment or complex anatomy [32,33,34]. Our findings reinforce the notion that the benefits of CR may extend to selected NSTE-ACS patients, especially those who are hemodynamically stable and able to tolerate multivessel intervention.

The decision to pursue CR in NSTE-ACS should be individualized, balancing potential ischemic benefit with procedural risks. Our findings suggest that in hemodynamically stable patients, CR may offer clinical benefits in reducing recurrent ischemic events and improving cardiac function. These results support a more proactive revascularization strategy in appropriate patients and highlight the need for structured post-discharge monitoring for cardiac recovery.

Several limitations warrant consideration: the retrospective, non-randomized design, although we used multivariate analysis to adjust for baseline differences, may have generated residual confounding that cannot be excluded. This was a single-center study, which limits generalizability, especially to centers with different interventional practices or patient populations. The smaller size of the IR group may have limited statistical power to detect differences in secondary outcomes. The treatment allocation to CR or IR was determined by operator judgment rather than random assignment, introducing potential selection bias. Although multivariable adjustment was applied, unmeasured confounders, such as lesion complexity, operator experience, or frailty could have influenced both treatment choice and clinical outcomes. The identification of the culprit lesion was based on clinical presentation and angiographic findings, without systematic use of advanced intracoronary imaging such as intravascular ultrasound or optical coherence tomography. This could have led to misclassification, particularly in patients with MVD where multiple unstable plaques may coexist. Physiologic lesion assessment using fractional flow reserve was not systematically performed for non-culprit lesions, meaning that some lesions may have been revascularized without functional significance, or conversely, significant lesions may have been left untreated in the IR group. Imaging variability may have been present because LVEF was assessed using echocardiography, which may be subject to inter-observer variation.

## 5. Conclusions

In this cohort of hemodynamically stable NSTE-ACS patients with MVD, CR was associated with lower rates of ischemic readmissions and improved left ventricular function at discharge, without a significant increase in length of hospital stay. These findings provide support for a more comprehensive revascularization approach in carefully selected patients and emphasize the need for prospective randomized trials specifically focused on NSTE-ACS.

## Figures and Tables

**Table 1:** Criteria for patients’ enrollment in the study.

Inclusion Criteria	Exclusion Criteria
Age ≥ 18 years	Cardiogenic shock or hemodynamic instability
Diagnosis of NSTE-ACS, defined according to contemporary guidelines (including both unstable angina and non-ST-elevation myocardial infarction) [3]	Prior coronary artery bypass grafting
Presence of multivessel coronary artery disease (≥2 major epicardial vessels with ≥70% stenosis)	Presentation with STEMI
Hemodynamic stability at the time of PCI (systolic blood pressure > 90 mmHg without inotropes or mechanical support)	Severe valvular heart disease or other structural heart disease requiring intervention
Underwent PCI during the index hospitalization	Missing data on key clinical outcomes or procedural details

NSTE-ACS, Non-ST-segment-elevation acute coronary syndrome; STEMI, ST-segment-elevation myocardial infarction; PCI, Percutaneous coronary intervention.

**Table 2:** Statistical analysis design.

Variable Type	Data Presentation	Statistical Test/Method
Continuous variables	Mean ± standard deviation or median (interquartile range), depending on distribution	Student’s *t*-test or Mann–Whitney U test
Categorical variables	Frequencies and percentages	Chi-square test or Fisher’s exact test
Primary composite outcome	Time-to-event data	Kaplan–Meier survival analysis and log-rank test
Hazard estimation	HRs with 95% CIs	Cox proportional-hazards regression, unadjusted and adjusted for covariates
Software	—	SPSS version 25 (IBM Corp., Armonk, NY, USA) [20]
Statistical significance		*p*-value < 0.05

HRs, hazard ratios; CIs, confidence intervals.

**Table 3:** Baseline features of the included cohort.

Parameter	CR Group (*n* = 218)	IR Group (*n* = 64)	*p* Value
Age, years	66.17 ± 9.59	67.16 ± 9.75	0.89
Sex (male) *n* (%)	144 (66.05)	34 (53.12)	0.01
Hypertension *n* (%)	148 (67.89)	53 (82.81)	0.02
Diabetes *n* (%)	108 (49.54)	44 (68.75)	0.004
Hypercholesterolemia *n* (%)	150 (68.81)	37 (57.81)	0.11
Current smoker *n* (%)	148 (67.89)	53 (82.81)	0.02
CKD *n* (%)	34 (15.60)	20 (31.25)	0.01
Atrial fibrillation *n* (%)	46 (21.10)	16 (25)	0.51
Serum creatinine mg/dL	0.89 (0.79–1.07)	0.92 (0.8–1.1)	0.57
Troponin, ng/L	1386 (405–2412)	1059.5 (472.5–2366.5)	0.27
GRACE risk score	114 ± 26	113 ± 23	0.19
SBP, mmHg	144 ± 19	149 ± 24	0.10
DBP, mmHg	85 ± 11	86 ± 12	0.63
Heart rate, beats/min	78 ± 17	84 ± 16	0.83
LVEF at admission, %	41 ± 8	43 ± 8	0.40

CR, complete revascularization; IR, incomplete revascularization; CKD, chronic kidney disease; GRACE, Global Registry of Acute Coronary Events; SBP, systolic blood pressure; DBP, diastolic blood pressure; LVEF, left ventricular ejection fraction. Values are *n* (%), median (interquartile range), or mean ± SD.

**Table 4:** Procedural characteristics of the studied population.

Parameter		CR Group (*n* = 218)	IR Group (*n* = 64)	*p* Value
Radial access *n* (%)		210 (96.33)	62 (96.88)	0.83
Femoral access *n* (%)		8 (3.67)	2 (3.12)	0.30
Culprit lesion	LM *n* (%)	26 (11.97)	7 (10.94)	0.66
	LAD *n* (%)	178 (81.65)	52 (81.25)	0.88
	LCX *n* (%)	164 (75.23)	44 (68.75)	0.30
	RCA *n* (%)	132 (60.55)	52 (81.25)	0.001
Clopidogrel *n* (%)		88 (40.37)	40 (62.50)	0.002
Ticagrelor *n* (%)		130 (59.63)	24 (37.50)	0.002

CR, complete revascularization; IR, incomplete revascularization; LM, left main coronary artery; LAD, left anterior descending coronary artery; LCX, left circumflex coronary artery; RCA, right coronary artery.

**Table 5:** The incidence of events in the studied population.

Parameter	6 Months			12 Months		
	CR Group (*n* = 218)	IR Group (*n* = 64)	*p* Value	CR Group (*n* = 218)	IR Group (*n* = 64)	*p* Value
Primary outcome *n* (%)	24 (11)	26 (40.63)	<0.001	48 (22.02)	44 (68.75)	<0.001
All-cause death *n* (%)	8 (3.67)	6 (9.38)		18 (8.26)	8 (12.5)	0.04
Cardiac death *n* (%)	3 (1.38)	6 (9.38)		7 (3.21)	6 (9.38)	<0.001
Unstable angina *n* (%)	8 (3.67)	12 (18.75)		14 (6.42)	22 (34.38)	
Myocardial infarction *n* (%)	6 (2.75)	14 (21.88)	0.001	18 (8.26)	20 (31.25)	
Stroke *n* (%)	6 (2.75)	0	0.18	8 (3.67)	2 (3.13)	0.67

CR, complete revascularization; IR, incomplete revascularization; Primary outcome, composite of all-cause death, cardiac death, and ischemic readmissions due to unstable angina, myocardial infarction, or stroke.

**Table 6:** LVEF changes and length of hospital stay.

Parameter Mean ± SD	CR Group (*n* = 218)	IR Group (*n* = 64)	*p* Value
LVEF at discharge, %	45 ± 6	43 ± 8	0.059
LVEF improvement, %	5 ± 3.8	0 ± 2.7	<0.001
Length of hospital stay, days	5.3 ± 2.9	5.8 ± 1.9	0.88

CR, complete revascularization; IR, incomplete revascularization; LVEF, left ventricular ejection fraction; SD, standard deviation.

**Table 7:** Correlation of different variables with the presence of the primary outcome, at 12 months.

Parameter		Primary Outcome		*p* Value
		Present *n* = 92 (32.6%)	Absent *n* = 190 (67.4%)	
Age, years		66.77 ± 9.12	66.22 ± 9.87	0.48
Sex (male)		50 (54.35)	128 (67.37)	0.03 *
Current smoker		38 (41.30)	80 (42.10)	0.79
Hypertension		73 (79.35)	128 (67.37)	0.03 *
Diabetes mellitus		60 (65.22)	90 (47.37)	0.005 *
Hypercholesterolemia		64 (69.57)	123 (64.74)	0.42
Atrial fibrillation		24 (26.08)	38 (20)	0.24
CKD		20 (21.74)	34 (17.89)	0.13
SBP, mmHg		148 ± 21	144 ± 20	0.65
DBP, mmHg		85 ± 12	86 ± 11	0.23
Heart rate		80 ± 12	79 ± 19	0.49
Troponins ng/L		964 (320.5–2416.5)	918.5 (369.5–2349)	0.976
GRACE risk score		112.5 ± 22	114.2 ± 27	0.52
LVEF admission, %		41 ± 10	43 ± 8	0.04 *
LVEF discharge, %		43 ± 8	46 ± 6	0.01 *
LVEF improvement, %		2.4 ± 2	3.9 ± 3	0.20
Culprit lesion	LM	7 (7.61)	26 (13.68)	0.002 *
	LAD	76 (82.61)	154 (81.05)	0.40
	ACX	71 (77.17)	137 (72.11)	0.06
	ACD	62 (67.39)	122 (64.21)	0.27
Clopidogrel		50 (54.35)	78 (41.05)	0.03 *
Ticagrelor		42 (45.65)	112 (58.95)	0.03 *
CR		48 (52.17)	170 (89.47)	<0.001 *

Values are *n* (%), mean ± SD, or median (interquartile range); primary outcome, composite of all-cause death, cardiac death, and ischemic readmissions due to unstable angina, myocardial infarction, or stroke; CKD, chronic kidney disease; SBP, systolic blood pressure; DBP, diastolic blood pressure; GRACE, Global Registry of Acute Coronary Events; LVEF, left ventricular ejection fraction; LM, left main coronary artery; LAD, left anterior descending coronary artery; LCX, left circumflex coronary artery; RCA, right coronary artery; * indicating *p* < 0.05.

## Data Availability

More data are available per request at the first author.

## Abbreviations

The following abbreviations are used in this manuscript:

CKD	Chronic kidney disease
CR	Complete revascularization
IR	Incomplete revascularization
MACE	Major adverse cardiovascular events
MVD	Multivessel disease
NSTE-ACS	Non-ST-segment elevation acute coronary syndrome
NSTEMI	Non-ST-elevation myocardial infarction
PCI	Percutaneous coronary intervention
STEMI	ST-segment elevation myocardial infarction
GRACE	Global Registry of Acute Coronary Events
SBP	Systolic blood pressure
DBP	Diastolic blood pressure
LVEF	Left ventricular ejection fraction
LM	Left main coronary artery
LAD	Left anterior descending coronary artery
LCX	Left circumflex coronary artery
RCA	Right coronary artery
HRs	Hazard ratios
CIs	Confidence intervals
